# Diversity of layer 5 projection neurons in the mouse motor cortex

**DOI:** 10.3389/fncel.2013.00174

**Published:** 2013-10-16

**Authors:** Manfred J. Oswald, Malinda L. S. Tantirigama, Ivo Sonntag, Stephanie M. Hughes, Ruth M. Empson

**Affiliations:** ^1^Department of Physiology, Brain Health Research Centre, Otago School of Medical Sciences, University of OtagoDunedin, New Zealand; ^2^Department of Biochemistry, Brain Health Research Centre, Otago School of Medical Sciences, University of OtagoDunedin, New Zealand

**Keywords:** electrophysiology, morphology, corticospinal, corticothalamic, corticocortical, corticostriatal, cluster analysis

## Abstract

In the primary motor cortex (M1), layer 5 projection neurons signal directly to distant motor structures to drive movement. Despite their pivotal position and acknowledged diversity these neurons are traditionally separated into broad commissural and corticofugal types, and until now no attempt has been made at resolving the basis for their diversity. We therefore probed the electrophysiological and morphological properties of retrogradely labeled M1 corticospinal (CSp), corticothalamic (CTh), and commissural projecting corticostriatal (CStr) and corticocortical (CC) neurons. An unsupervised cluster analysis established at least four phenotypes with additional differences between lumbar and cervical projecting CSp neurons. Distinguishing parameters included the action potential (AP) waveform, firing behavior, the hyperpolarisation-activated sag potential, sublayer position, and soma and dendrite size. CTh neurons differed from CSp neurons in showing spike frequency acceleration and a greater sag potential. CStr neurons had the lowest AP amplitude and maximum rise rate of all neurons. Temperature influenced spike train behavior in corticofugal neurons. At 26°C CTh neurons fired bursts of APs more often than CSp neurons, but at 36°C both groups fired regular APs. Our findings provide reliable phenotypic fingerprints to identify distinct M1 projection neuron classes as a tool to understand their unique contributions to motor function.

## INTRODUCTION

The primary motor cortex (M1) plays a central role in controlling movement execution through its long-range projections to the spinal cord. Activity in M1 correlates well with actual movement trajectories (Kakei et al., 1999; Buch et al., 2010; Vargas-Irwin et al., 2010; Pearce and Moran, 2012) whilst activity in premotor and supplementary motor areas that also project to the spinal cord (Nudo and Masterton, 1990; Dum and Strick, 1991) typically occurs during movement preparation.

Aside from the direct corticospinal (CSp) projections to the spinal cord, M1 makes direct corticofugal projections to target the thalamus, zona incerta and brainstem motor centers (Gao and Zheng, 2004; Mao et al., 2011) and collaterals from these projections also innervate the ipsilateral striatum (Donoghue and Kitai, 1981; Cowan and Wilson, 1994; Reiner et al., 2003). Corticocortical projections (short and long-range) include connections with other cortical regions in the same hemisphere and homotopic areas in the contralateral M1 (McGuire et al., 1991; Aronoff et al., 2010; Mao et al., 2011). Striata in both hemispheres are another major target of M1 projection neurons with the likelihood that they also send collaterals to the contralateral cortex (Wilson, 1987; McGeorge and Faull, 1989; McGuire et al., 1991).

Neurons projecting to all these targets are found in layer 5 of M1. Despite their different projection targets and acknowledged heterogeneity (Tseng and Prince, 1993; Cho et al., 2004a,b; Gao and Zheng, 2004) they are generally described as commissural or corticofugal projection neurons with electrophysiological, synaptic and morphological properties similar to those seen in neocortex (Chagnac-Amitai et al., 1990; Mason and Larkman, 1990; Schubert et al., 2006) and frontal, sensory and motor cortices (Kasper et al., 1994; Christophe et al., 2005; Morishima and Kawaguchi, 2006; Le Be et al., 2007; Anderson et al., 2010; Groh et al., 2010; Sheets et al., 2011; Kiritani et al., 2012; Suter et al., 2012). Given the diversity of projection targets for the motor cortex and the growing detail of cellular differences in other cortices (Hattox and Nelson, 2007; Otsuka and Kawaguchi, 2008, 2011; Brown and Hestrin, 2009; Schmidt et al., 2012), a separation based upon two broad projection neuron types in M1 seems limiting and dated. Therefore, we aim here to expand the basis for diversity of projection neurons in M1 using retrograde targeting approaches, detailed morphological and electrophysiological parameters and unsupervised cluster analysis.

We find genuine differences amongst layer 5 neuron phenotypes in M1 based upon their projection target and beyond those already established for other cortical regions. The knowledge of several key parameters is sufficient to reliably assign a neuron to one of at least four specific subtypes in layer 5 that match well with the four principal projection targets. This useful phenotypic fingerprinting approach significantly adds to our understanding of diversity within layer 5 of M1 and provides a useful tool to aid further analysis of this central motor structure.

## MATERIALS AND METHODS

### ANIMALS

All procedures were approved by the University of Otago Animal Ethics Committee in accordance with the New Zealand Animal Welfare Act 1999. Male Swiss-Webster mice were used for all experiments.

### RETROGRADE LABELING

Stereotaxic injections of retrograde tracers [cholera toxin subunit B – Alexa Fluor 647 conjugate; (CTB, 0.35% (w/v) in saline) or tetramethylrhodamine conjugated 10 kDa dextran (fluoro-Ruby, 5% (w/v) in saline), both from Molecular Probes] were performed at postnatal day (P) 18–21. Animals were deeply anesthetized with ketamine (70 mg/kg), medetomidine (0.5 mg/kg) and atropine (0.05 mg/kg) delivered subcutaneously. Lidocaine (4 mg/kg) was applied subcutaneously as a local analgesic and Carprofen (5 mg/kg) administered as a general analgesic at the start of the surgery. A mild flow of oxygen (0.1 L/min) provided a respiratory aid throughout the surgery.

Forelimb and hindlimb CSp neurons were labeled selectively via retrograde cholera toxin subunit B – Alexa Fluor 647 conjugate (as above) injections into the cervical or lumbar enlargement, respectively. To perform a laminectomy, 2–3 vertebrae were exposed at the C3/C4 or Th13/L1 level and the caudal half of either the C3 or Th13 vertebra lamina removed with fine tweezers and the dura pierced. A glass pipette (internal tip Ø of ~25 μm, pulled from graduated 5 μl glass capillaries, Vitrex Modulohm) positioned 0.45 mm lateral from the midline to a depth of 0.7 mm below the pia allowed delivery of 1 μl of tracer at a rate of 200 nl/min by pressure ejection. The pipette remained in place, undisturbed, for 3 min before stepwise retraction over a further 2–7 min period before wound closure and reversal of anesthesia by subcutaneous administration of antisedan (2.5 mg/kg).

To access forebrain structures a 35 or 36G beveled Nanofil needle (World Precision Instruments, bevel facing posteriorly) was lowered through a hole in the skull to one of the following stereotaxic injection coordinates (A-P, anterior-posterior to Bregma; ML, medio-lateral to midline; DV, dorso-ventral from the pia, in mm): motor cortex, left hemisphere, 0 to 0.6 AP, 1.1 to 1.3 ML, 0.7 DV; Striatum, left hemisphere, 0.4 AP, 2.2 ML, 2.2 DV; Ventrolateral thalamic nucleus, right hemisphere, -1.4 AP, 1.0 ML, 3.5 DV. CTB (0.2–0.4 μl, 0.35% (w/v) in saline) was pressure injected at 100 nl/min using a Micro syringe pump and a 10 μl Nanofil syringe (World Precision Instruments). Needle retraction, suturing of the incision, and anesthesia reversal were as above.

### VGLUT2 IMMUNOHISTOCHEMISTRY

Male mice, P25–33, were deeply anesthetized with sodium pentobarbital (150 mg/kg, i.p.) and intracardially perfused with 4% paraformaldehyde in 0.1 M sodium phosphate buffered saline (PBS, pH 7.4). Fifty micron thick sections of forebrain were cut on a freezing microtome with the coronal plane tilted 10° rostrally. Sequential brain sections for immunohistological analysis were stored in cryoprotectant (15% sucrose, 30% ethylene glycol, 0.05 M PBS, pH 6.8) at -20°C in 96-well plates for 2–14 days. Serial sections matched for distance from Bregma (0.5 mm interval) were rinsed first in PBS, then PBS containing 0.25% Triton X-100 (PBS-T), and incubated with a rabbit polyclonal antibody to VGLUT2 (Synaptic Systems, affinity purified, 2 μg/ml) in PBS-T containing 5% goat serum for 72 h at 4°C. After 3 × 10 min washes with PBS-T, the sections were incubated with Alexa Fluor 555-conjugated goat anti-rabbit secondary antibody (Invitrogen, 1:500) in PBS-T containing 1% goat serum for 3 h at room temperature. After washing, sections were rinsed in PBS-T and PBS, mounted on glass slides and coverslipped using Vectashield mounting medium (Vector Labs).

Serial sections with retrograde labeled neurons were mounted without further processing after cutting. Sections were observed by epifluorescence microscopy (BX51, Olympus) using 545–580 nm excitation and 610 nm long pass emission.

The primary motor cortex was identified with the aid of a stereotaxic atlas (Paxinos and Franklin, 2001). The upper layer 5 boundary with layer 2/3 was determined based on the stronger VGLUT2 immunoreactivity of lower layer 2/3 compared to upper layer 5. Layer 5 was further subdivided based on the absence or presence, respectively, of retrograde labeled somata from the spinal cord into layer 5A (L5A) with very weak VGLUT2 immunoreactivity, and layer 5B (L5B) with distinct VGLUT2 immunoreactivity. The lower layer 5 boundary with layer 6 is an estimate based on the distribution of retrograde labeled somata from the spinal cord above this boundary in L5B, and the weaker VGLUT2 immunoreactivity in layer 6 compared to L5B.

### SLICE PREPARATION

Following a survival period of 3–14 days (P23–32), acute coronal slices of the right hemisphere spanning the motor cortex were prepared. The animals were sedated by exposure to CO_2_ gas, decapitated, the brain immediately removed and submerged in ice-cold cutting solution (in mM: 87 NaCl, 75 sucrose, 2.5 KCl, 6 MgCl_2_, 0.5 CaCl_2_, 25 Glucose, 25 NaHCO_3_, 1.25 NaH_2_PO_4_) saturated with 95% O_2_ and 5% CO_2_. The coronal blocking cut was tilted 10° rostrally in the plane of the apical dendrites within slices of the targeted motor cortex, and slices (300 μm) cut using a vibratome (VTS1000S, Leica). The slices were transferred to artificial cerebrospinal fluid (ACSF) containing (in mM) 126 NaCl, 3 KCl, 1 NaH_2_PO_4_, 2 MgSO_4_, 2 CaCl_2_, 25 NaHCO_3_, 15 glucose, saturated with 95% O_2_ and 5% CO_2_, and maintained initially at 35°C for 30 min and then at room temperature for at least another 30 min before recording.

### ELECTROPHYSIOLOGICAL RECORDING

Slices were transferred to the recording chamber of an upright microscope and retrograde labeled neurons visualized using epifluorescence (640 ±7 nm excitation, 676 ±15 nm emission) and IR-DIC optics (Eclipse FN1, Nikon). Layer 5 was identified based on the retrograde labeling profile of CSp and CTh neurons and the presence of larger neuronal somata compared to layers 2/3 or 6. Soma location in layer 5 of M1 was verified *post hoc* by biocytin labeling with reference to laminar 5 boundaries determined with VGLUT2 immunostaining (see above). Glass electrodes had a resistance of 5–7 MΩ when filled with (in mM) 10 KCl, 10 Na-phosphocreatine, 110 K-gluconate, 10 HEPES, 4 Mg-ATP, 0.3 Na_2_-GTP, 0.2% biocytin, adjusted to pH 7.3 and 295 mOsm with sucrose. Whole cell patch-clamp recordings were obtained using a Multiclamp 200B amplifier (Molecular Devices), filtered at 4–10 kHz and sampled at 20 kHz with a 1401 plus digitizer (CED) and Signal acquisition software (CED). Pipette capacitance was neutralized and the series resistance compensated with the bridge balance circuit in current clamp mode. No liquid junction potential correction was applied. Input resistance, resting membrane potential and access resistance, typically less than 20 MΩmega, were carefully monitored and cells were excluded if any of these changed by >20% during the recording period. Slices were continually perfused with ACSF supplemented with 2,3,4-tetrahydro-7-nitro-2,3-dioxoquinoxaline-6-carbonitrile disodium, 10 μM (CNQX, Abcam Biochemicals), D-2-amino-5-phosphonopentanoic acid, 50 μM (D-APV, Abcam Biochemicals), and picrotoxin, 50 μM (Sigma-Aldrich) to block fast synaptic input. Temperature was controlled with an inline heating system (TC-324B, Warner Instruments) set to either 26 or 36°C.

### BIOCYTIN LABELING

After electrophysiological recordings the slice was fixed in 4% paraformaldehyde overnight at 4°C and then stored in PBS, or cryoprotected in 30% sucrose and stored at -80°C. Visualization of biocytin-filled neurons used two methods. Slices were rinsed three times in PBS and permeabilised in PBS containing 0.3% Triton X-100 for 2–4 h at room temperature, then incubated for another 2–3 h in either an avidin-biotin-peroxidase solution (Vectastain ABC elite kit, Vector Laboratories) or with streptavidin Alexa 568 (2 μg/mL, Invitrogen). Those processed with ABC used diaminobenzidine (DAB) solution (ImmPACT DAB peroxidase substrate kit, Vector Laboratories). Slices were prepared and mounted as above. DAB-processed slices were air dried after mounting, dehydrated through incubation in a series of increasing ethanol concentrations, equilibrated in xylene and coverslipped with DPX. To correct for possible shrinkage effects, three Alexa 568 labeled neurons were imaged immediately after mounting (see below) and the slices subsequently incubated in avidin-biotin-peroxidase solution and processed for DAB labeling as described.

### MORPHOLOGY RECONSTRUCTION

Diaminobenzidine-processed neurons were manually traced with the aid of a camera lucida drawing tube attachment on an Olympus BX-50 microscope with a 40× objective, and then digitized. For slices processed with streptavidin Alexa 568, fluorescence images were acquired on an Olympus BX51 microscope using a 20×/0.75 NA objective, 545–580 nm excitation and 610 nm long pass emission. Sequential images were taken at ~10 μm *z*-step intervals to capture the depth of the dendritic tree. Stacks of 20–60 images were flattened by calculating the maximal intensity of the z-stack (ImageJ) and the resulting tiles merged into one image using Adobe Photoshop by manually aligning common landmarks in each tile. Neuronal morphology was then traced manually using the freely available ImageJ plugin NeuronJ (Meijering et al., 2004). The axon was usually not reconstructed beyond approximately 200 μm from the soma.

### MORPHOLOGY ANALYSIS

Cellular location in M1 and the rostro-caudal position relative to bregma used the mouse brain atlas (Paxinos and Franklin, 2001). We defined cortical depth as the distance from the pial boundary through the center of the soma to the border of layer 6 and white matter. Cell depth was measured by extending a line from the center of the soma to the outermost boundary of the pia in line with the axis of the apical dendrite. Pial cell depth was normalized to the cortical depth. Apical dendrite height was measured in a vertical line from the base of the dendritic shaft to the highest point in the tuft. The dendritic tuft height and width were measured as the rectangular dimensions defined by the point at which the apical dendrite bifurcated to the highest and widest points in the tuft. We used a 60×/1.35 NA oil immersion objective to image the soma and proximal dendrites. Soma size was measured as the average of height and width and a soma shape factor also incorporated from the ratio of these two values. Shaft width was measured 20 μm from the center of the soma. Morphology measurements from DAB-processed neurons were shrinkage corrected by a factor of 0.75 determined from the average ratio of pial depth measurements taken from three neurons that were imaged and traced first using fluorescence detection, and then traced again after DAB-processing with the camera lucida method. Morphological data was not otherwise corrected for potential tissue shrinkage effects due to the fixation and histological labeling procedure.

We measured dendritic complexity using a 2-dimensional Sholl analysis on dendrites of reconstructed neurons (ImageJ Sholl analysis plugin). Circles incremented with a radial step size of 10 μm centered around the soma. Radial distances were either normalized to cortical depth or depth of soma from the pia, then multiplied again by the average cortical width or soma depth of all neurons, respectively, and averages of the Sholl counts calculated at 10 μm bins.

### DATA ANALYSIS

Signal CFS data files were converted for analysis to Matlab (7.10) data files (The MathWorks) based on scripts from the Matlab CFS library supplied by James Colebatch (), and the data analyzed using custom-written scripts. Input resistance (Ri) was calculated from the average peak voltage deflection in response to 500 ms long current steps of -20 or -25 pA repeated 20 times, relative to the preceding 50 ms baseline. The membrane time constant τ _m_ was determined from the slowest exponential term derived by fitting a double exponential function (Eq. 1) to the curve of the average membrane potential obtained in response to the negative current steps. The nlinfit Matlab function using the Levenberg-Marquardt algorithm for nonlinear least square fits obtained the best fit between *t* = 0 and the time of the peak membrane deflection.

1 $$V(t)={ale}^{-t/\tau 1}+a2\,\;e^{-t/\tau 2}+a3\,\;$$

The hyperpolarisation-activated cation current (*I*_H_) was estimated from the sag in the membrane potential (defined by the difference between the peak potential and the steady state potential during the final 80 ms of the 1 s long current step) in response to a series of current steps from -200 to -40 pA. Individual traces were smoothed with a Savitzky–Golay filter of 0.5 ms window width to allow an accurate measure of the peak potential. Sag potentials were normalized to the respective peak voltage deflection from the resting membrane potential and the average of the normalized sag potentials used as an estimate of *I*_H_ for each neuron. The magnitude of the post-train afterhyperpolarisation (AHP) was determined from the maximum amplitude (defined as the difference between the minimal potential averaged over a 1 ms period, compared with the baseline) detected in response to consecutive 1 s long depolarising current steps (up to 750 pA for commissural neurons, and 1000 pA for corticofugal neurons).

Action potential (AP) parameters were measured from the first AP evoked during a 1 s long current ramp generated by consecutively increasing the final value in the ramp by 10 pA. We applied a threshold of 1.2 V/s to determine the membrane potential at which the AP was initiated, and AP amplitude was defined as the difference between the peak and the threshold of the AP. The amplitude of the medium AHP (mAHP) following the AP was calculated by subtracting AP threshold from the minimum membrane potential detected within a 100 ms period from AP onset at AP threshold. AP half width was calculated from the difference in time between sample points found nearest to the 50% mark of the AP amplitude. Maximum rise and decay kinetics of the AP were defined as the maximum and minimum values of the first derivative of the membrane potential computed using a difference procedure:$dV(t)/dt=[V(t_{n})-V(t_{n-1})]/(t_{n}-t_{n-1})$

The spike firing response to a series of 1 s long current steps increasing by 50 pA in consecutive steps (up to 750 pA for commissural neurons, and 1000 pA for corticofugal neurons) was determined as follows. APs were detected using a threshold criterion on the first derivative of the membrane potential smoothed with a Savitzky–Golay filter of 0.8 ms window width. The time of each AP maximum was used to calculate interspike interval (ISI) times; 1/ISI provided the instantaneous firing frequency. The mean instantaneous firing frequency was computed for episodes containing >5 APs. AP adaptation was determined for traces with >7 APs after normalizing all ISIs to the third ISI in each trace and computing the slope constant of a linear regression of the normalized ISIs against ISI number. The first two ISIs were excluded from the regression analysis. Burst-like episodes were avoided by excluding episodes that contained ISIs lasting more than five times that of the third ISI. The spike adaptation index was defined as the mean of three slope constants from trials with a mean instantaneous firing frequency closest to 15 Hz over the range of ISIs used for the regression. An adaptation index close to zero represents regular firing, and an index smaller or greater than zero indicates spike frequency acceleration or slowing, respectively. Neurons that showed a decrease in the mean instantaneous firing rate over two or more steps of increasing current injection from firing threshold were defined as intrinsically bursting.

The total AP count in response to each 1 s long depolarization provided the mean firing frequency (*Fqz*). The nlinfit Matlab function was used to obtain the least square fit of a sigmoidal function (Eq. 2) to the mean spiking frequency data for successive current steps (*I*_steps_) from *I*_step_ = 0 to the *I*_step_ that evoked the maximum AP frequency response.

2 $$Fqz(I_{step})=k1/(1+e^{(I_{50}-I_{step})/k2})\;\;\;\;\;\;\;\;\;\;\;\;\;\;\;\;\;\;\;\;\;\;$$

The computed constants k1 and *I*_5__0_ respectively represent the maximum AP frequency and the step current generating an AP output at 50% of the maximal frequency of the sigmoidal fit to the data. The gain of the firing frequency increase was defined by the slope of a linear regression performed on the fitted values predicted by the sigmoid fit over a frequency range of 25–75% of the maximal firing frequency. The sigmoid fit was selected only if the sum of the squared residuals was smaller than the sum of the squared residuals obtained from a linear regression performed on the same data set. *I*_50_ threshold and maximum frequency were not computed for neurons with spike frequency responses best fit with a linear model, and instead the slope constant of the overall linear regression was used.

### STATISTICAL ANALYSIS

Statistical analyses were performed in Prism 5 (GraphPad Software) or the Statistics Toolbox in Matlab (version 7.10). The D’Agostino-Pearson omnibus test was used to test data for normality and non-parametric tests used if one or more of the data subgroups did not follow a normal distribution. To test for differences in cellular properties between cell types, either a one-way ANOVA followed by Tukey’s *post hoc* test or the non-parametric Kruskal-Wallis test followed by Dunn’s *post hoc* analysis was used. The effect of recording temperature on cellular properties for different cell types was tested on neurons that were recorded at both temperatures using the anovan Matlab function followed by a *post hoc* test for cell-type differences using Tukey’s honestly significant difference criterion. To compare the number of Sholl intersections between cell types, radial distances were either normalized by cortical depth or depth of soma from pia, and a two-way ANOVA with Bonferroni’s *post hoc* test used. Group data are presented as means ±SEM.

We entered a total of 24 parameters into an unsupervised cluster analysis performed in Matlab, using Euclidean distances and Ward’s method for linkage of neurons: RMP, Ri, membrane time constant, sag, Bregma level, AP threshold, AP amplitude, AP half-width, AP maximum rise and decay, mAHP, post-train AHP, pial cell depth normalized to cortical width, soma size, dendritic shaft width, tuft height and width, tuft origin and apical dendrite height normalized to cell depth, adaptation index, maximum mean instantaneous firing frequency, maximum mean firing rate, input current at 50% max frequency, and maximum firing frequency gain. Missing values were replaced with the Matlab knnimpute function based on a euclidean distance match to the nearest neighbor, and the data was standardized to a single scale by z-scoring each variable. For the principal component (PC) analysis of the same variables, data was further normalized before z-scoring by taking the square root (tuft height and maximum frequency gain), the square root after subtracting the minimum value (RMP, sag, shaft width and soma depth), the natural logarithm (Ri, membrane time constant, AP half-width and max decay and I_50% max frequency_), the reciprocal (maximum mean firing rate), squaring (AP amplitude and tuft origin), or squaring after subtracting the minimum value (adaptation index).

## RESULTS

**FIGURE 1:**
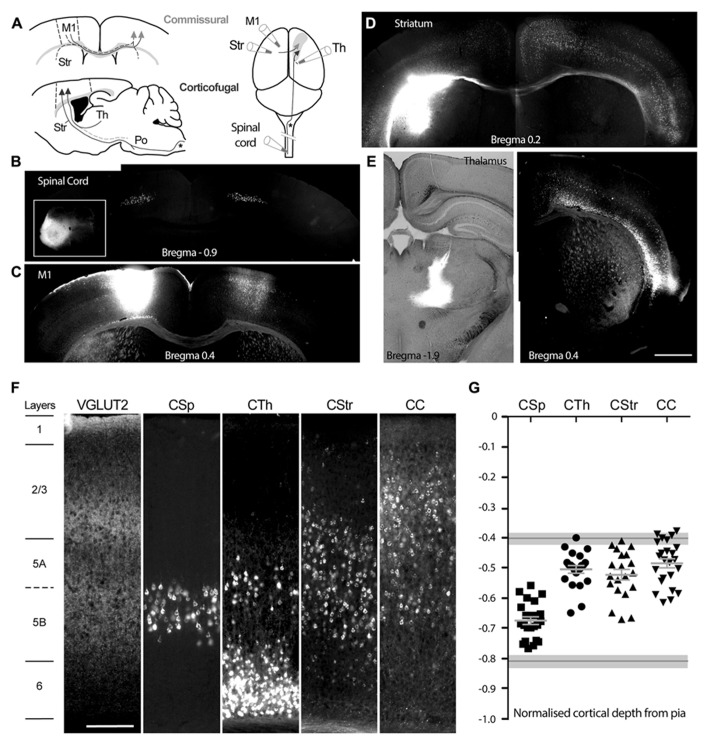
**Injection sites and profiles of retrogradely labeled neurons in mouse primary motor cortex, M1. (A)** Schematic of retrograde tracer injection sites targeting either commissural neurons projecting through the corpus callosum across hemispheres, or corticofugal neurons projecting ipsilaterally to the thalamus, brainstem, and spinal cord. M1 (borders delineated by the dashed lines) denotes the site for labeling of corticocortical neurons CC, in the contralateral hemisphere, see arrow heads; Str denotes the site for labeling of corticostriatal, CStr, neurons, these neurons also send collaterals across the midline to the contralateral cortex, dashed line with arrowheads. Spinal cord and Th denote sites for labeling of corticospinal, CSp and corticothalamic, CTh neurons respectively. Note that the corticospinal tract decussates in the medulla oblongata (shown by an asterisk *) and that axons projecting from the thalamus to M1 may be collaterals of neurons that project to the brainstem, e.g., pontine nuclei, Po, as shown by the dashed line, lower panel **(A)**. **(B)** Bilateral labeling of corticospinal neurons in M1 and S1 hindlimb regions following a unilateral cholera toxin B (CTB) injection into the lumbar spinal cord (inset). **(C)** Homotopic M1 labeling contralateral to the CTB injection site in M1. **(D)** Widespread cortical bilateral labeling following CTB injection into the dorsolateral striatum in the left hemisphere (contrast was enhanced for the right hemisphere). **(E)** Widespread labeling of layer 5 and 6 neurons following ipsilateral CTB injection into the ventrolateral thalamic nucleus. **(F)** Laminar profile of VGLUT2 immunoreactive terminals and distribution of the retrogradely labeled neurons in M1, from the different sites, in slices of M1 obtained from similar bregma levels. **(G)** Soma position of all individually patch-clamped retrograde labeled neurons normalized by cortical depth, aligned to the layer positions in **(F)**. Mean ±SEM for each projection neuron group are shown in light gray. Upper and lower layer 5 boundaries based on VGLUT2 immunoreactive terminal distribution are indicated by gray lines with light gray shading (mean ±SD). Scale bars: 1 mm (**E**, representative for **B–E**) and 200 μm **(F)**. Images obtained from representative 50 μm thick histologically processed sections.

We injected 46 mice with retrograde tracer at one of four known target regions of M1 (**Figure 1A**), the spinal cord and ventrolateral thalamus to label corticofugal (CSp and CTh) projection neurons, and the striatum or contralateral motor cortex to label commissural (corticostriatal, CStr and corticocortical, CC) projection neurons in M1 (**Figures 1B,D**). Lumbar spinal cord injections labeled projection neurons in M1 and the neighboring primary somatosensory cortex between bregma levels +0.3 and -1.5, with a skewed distribution weighted toward caudal levels. Cervical spinal cord injections resulted in more widespread labeling of agranular motor regions (Nudo and Masterton, 1990). Neurons labeled from cervical spinal cord were prominent in forelimb regions of M1 at more rostral levels but also extended to the caudal levels of M1 and associated somatosensory hindlimb regions. We sampled lumbar CSp neurons between bregma levels -1.3 and +0.3, and CSp neurons labeled from the cervical spinal cord between bregma levels -0.8 to +0.8. Retrograde tracer injections into the thalamus resulted in widespread labeling within the ipsilateral hemisphere (**Figure 1E**) and we sampled neurons between bregma levels -1.2 and +1.3. Commissural neurons projecting to the motor cortex originated predominantly in the contralateral motor cortex where they appeared in a homotopic pattern (**Figure 1C**) and we sampled these neurons between bregma levels -1.2 and +1.3. In contrast, neurons projecting to the dorsolateral striatum distributed widely across cortical regions in both hemispheres (**Figure 1D**) and we sampled these between bregma levels -1.3 and +0.5.

### LAYER BOUNDARIES

We assigned cortical layer boundaries between layer 1 and 2 based on the absence and presence of pyramidal neuron soma, and between layers 5a and 2/3 as well as layers 5b and 6 by the profile of vesicular glutamate transporter-2 (VGLUT2) immunoreactive fiber density (**Figure 1F**; Morishima et al., 2011; Hirai et al., 2012). The M1 layer 5a boundary with layer 2/3 occurred at a normalized depth of 0.40 ±0.02 from the pia (*n* = 3) and remained constant over all bregma levels. The lower layer 5 boundary with layer 6 was estimated at a pial depth of 0.81 ±0.02 (*n* = 3). CSp neurons were located in layer 5b and CTh neurons mostly more superficially in layer 5a as well as layer 6 (**Figure 1F**). CStr neurons occurred throughout layer 5 whereas CC neurons were most often found in upper layer 5 and lower layer 2/3.

In total we sampled 99 retrogradely labeled neurons from one of the four different target sites across bregma levels ±1.3. In order to compare the layer profile of each group of projection neurons (and since cortical depth decreases in the rostrocaudal direction, overall range of 934 to 1795 μm) we normalized the depth of each of their soma from the pia to the cortical depth (**Figure 1G**); note that these are approximate positions of the electrophysiologically targeted neurons in our dataset. The CSp neurons occupied the deepest position in layer 5 whilst commissural (CC and CStr) and CTh projection neurons located more superficially (**Figure 1G**; **Table 1**).

**Table 1:** Morphological characteristics of M1 layer 5 projection neurons.

	CSp (*n* = 19)^†^	CTh (*n* = 20)	CStr (*n* = 16)	CC (*n* = 23)	Statistics^‡^
Cell depth^∫^	0.67 ±0.01	0.51 ±0.02	0.52 ±0.02	0.48 ±0.01	a^3^, b^3^, c^3^
Soma size (μm)	19.7 ±0.6	18.5 ±0.6	15.4 ±0.4	16.1 ±0.7	b^3^, c^3^, d^2^, e^1^
Soma height/width ratio	1.47 ±0.04	1.46 ±0.05	1.42 ±0.06	1.29 ±0.04	c^1^, e^1^
Apical dendrite height^§^	0.97 (0.96, 0.98)	0.98 (0.97, 0.98)	0.94 (0.91, 0.96)	0.93 (0.85, 0.98)	b^1^, d^3^, e^2^
Tuft origin^§^	0.45 (0.35, 0.61)	0.55 (0.43, 0.63)	0.70 (0.63, 0.75)	0.68 (0.64, 0.75)	b^3^, c^3^, d^1^, e^2^
Tuft height (μm)	509 (315, 594)	283 (189, 362)	153 (128, 185)	140 (92, 185)	b^3^, c^3^, d^1^, e^3^
Tuft width (μm)	379 ±21	429 ±29	191 ±27	160 ±20	b^3^, c^3^, d^3^, e^3^

† CSp, corticospinal neurons; CTh, corticothalamic neurons; CStr, callosal corticostriatal neurons; CC, callosal corticocortical neurons.

‡ One-way ANOVA and Tukey’s *post hoc* test for multiple comparisons on normally distributed data (mean ±SEM), otherwise non-parametric Kruskal-Wallis test and Dunn’s *post hoc* analysis (median values with the 1st and 3rd quartile stated in brackets); a, CSp vs CTh; b, CSp vs CStr; c, CSp vs CC; d, CTh vs CStr; e, CTh vs CC; f, CStr vs CC; ^1^*p* < 0.05, ^2^*p* < 0.01, ^3^*p* < 0.001.

∫ Normalized to cortical width.

§ Normalized to cell depth from the pia.

### MORPHOLOGICAL CHARACTERISTICS

**FIGURE 2:**
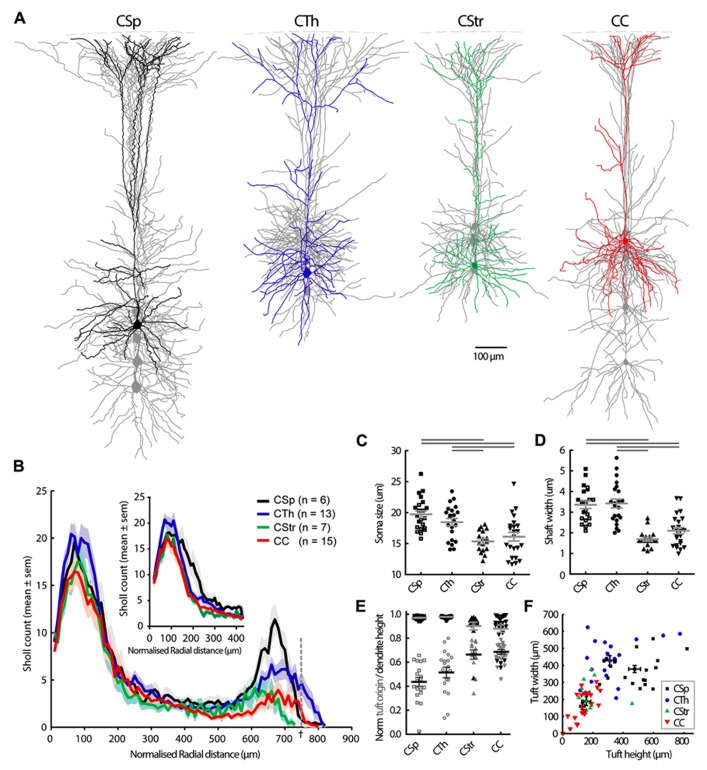
**Morphological characteristics of M1 layer 5 neurons grouped according to the projection target. (A)** Grouped overlays of 5 single reconstructed neurons aligned at the pia (dashed line). One cell is highlighted in each group. Axons are not shown. The CSp neurons shown were labeled from the lumbar spinal cord but cellular morphologies are representative also for cervical projecting CSp neurons. **(B)** Sholl analysis of dendritic complexity with increasing radial distance from the soma center. Sholl radii were normalized by soma depth from the pia and multiplied again by the average soma depth of all cells across all groups (†, the dashed line represents the sholl radius that equals soma depth from the pia), or by cortical depth and muliplied again by the average cortical depth (inset for basal dendrites only). Cell groups differed mainly in the distal apical tuft region (*p* < 0.001, two-way ANOVA). Soma size and shaft width (**C, D**, filled and open squares respectively for lumbar and cervical CSp neurons). Horizontal gray bars indicate group-wise differences at the 5% significance level (**C**, one-way ANOVA and Tukey’s *post hoc* test; **D**, Kruskal-Wallis test and Dunn’s *post hoc* analysis). Apical dendrite height (**E**, filled black symbols, gray lines for mean ±SEM) and tuft origin (open gray symbols, black lines for mean ±SEM). **(F)** Overall correlation of tuft width and height showing data from individual neurons and the mean ±SEM for each group.

We recovered morphological features of 78 biocytin-filled neurons and reconstructed the dendrites of 41 projection neurons and submitted these to Sholl analysis (**Table 1**; **Figure 2**). We did not observe any clear evidence for dye coupling between biocytin filled cells in any of the neuron types. The CSp neurons were the largest (**Figure 2C**) and those projecting to the lumbar spinal cord had larger soma and dendritic shafts than those labeled from the cervical spinal cord (see open versus closed squares in **Figure 2C**, mean values for soma size were 22 ±0.8 in lumbar cells compared with 18 ±0.4 in cervical; mean values for shaft widths were 4 ±0.2 in lumbar compared with 2.9 ±0.2 in cervical). Other morphological characteristics did not differ so we pooled lumbar and cervical CSp neurons in all further analysis.

Corticospinal and corticothalamic neurons both possessed soma and primary apical dendritic shafts and distal apical tufts that were larger and thicker than those of commissural projecting neurons (**Figures 2A,C,D,F**). The larger distal apical tufts also ramified more extensively in the superficial layer 1 close to the pia (**Figures 2A,E,F**; **Table 1**). The apical dendrites of CTh neurons had the widest lateral reach but CSp apical dendrites branched more and were more dense in superficial layers 1 and 2 (**Figure 2B**). In contrast the basal dendrites of CTh neurons branched most, an outcome that was most apparent when normalizing Sholl radial distance to cortical depth (**Figure 2B** inset). The latter analysis also revealed that the proximal dendrites of CSp neurons branched more extensively close to the soma (**Figure 2B** inset, 150–200 μm). This reflects their deeper laminar position and the prevalence of oblique dendrites emanating within layer 5 (**Figure 2A**). CC and CStr neurons exhibited very similar Sholl profiles. Differences in the soma shape of corticofugal and commissural neurons were less pronounced although CC neuron soma tended to be roundest and least elongated overall (**Table 1**).

### ACTION POTENTIAL FIRING RATE AND PATTERN

**FIGURE 3:**
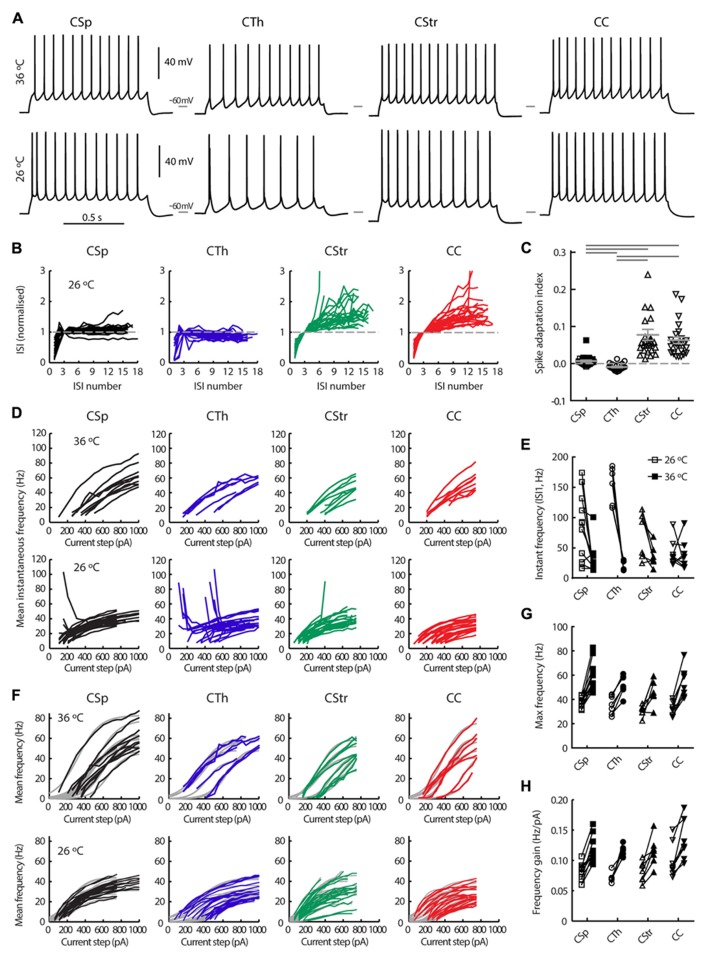
**Action potential firing behavior of M1 layer 5 neurons grouped by projection target. (A)** Firing responses of CSp, CTh, CStr and CC neurons during a 1 s long depolarising current injection recorded in the same neuron at 36 and 26°C. **(B)** Normalized inter spike interval (ISI) plots for all neurons in each group derived from episodes with a firing frequency closest to 15 Hz at 26°C. **(C)** The spike adaptation index, defined as the slope of the linear regression of the ISI plots in B, from the 3^rd^ ISI onward (**C**, means ±SEM for each group are shown in gray, filled and open squares respectively for lumbar and cervical CSp neurons where visible). Horizontal gray bars indicate group-wise differences of the Log normalized data (*p* < 0.001, one-way ANOVA and Tukey’s *post hoc* test). **(D)** Mean instantaneous frequency for five or more action potentials per current step for all neurons in each group. **(E)** Instantaneous frequency of the 1st ISI of the episode with a firing frequency closest to 15 Hz (paired recordings at 26 and 36°C). A two-way repeated measures ANOVA of the Log normalized data revealed that cell types fired at similar initial instantaneous frequencies (*p* = 0.62) but temperature had an effect on this measure selectively in CSp and CTh neurons (*p* < 0.01 and 0.001, respectively, Bonferroni’s *post hoc* analysis). **(F)** Mean firing frequency response to successively increasing depolarising current injections for each neuron at 26 and 36°C, and best sigmoidal or linear fits of the data (gray curves in background). Maximum mean frequency **(G)** and frequency gain **(H)** for all neurons at both temperatures.

**FIGURE 4:**
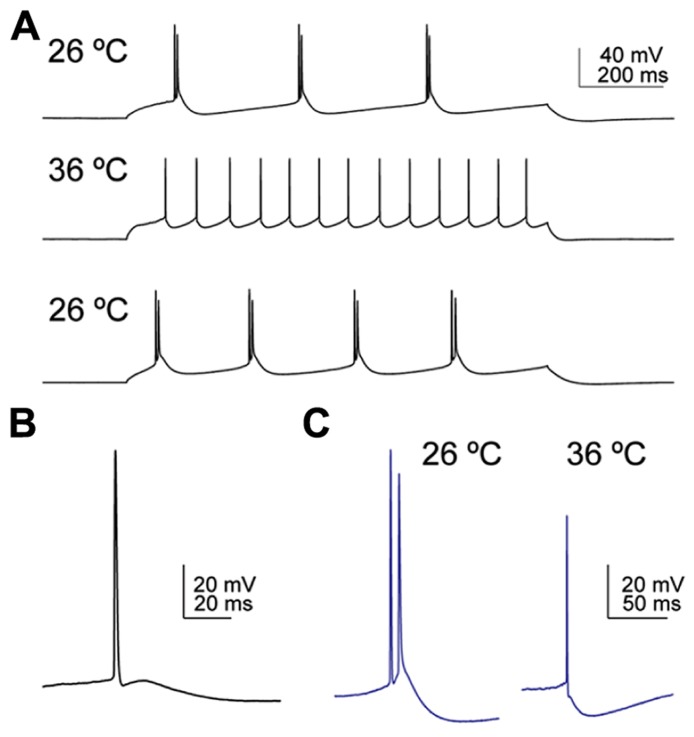
**Temperature influences the firing pattern and the depolarising afterpotential (DAP) following an action potential in corticofugal neurons. (A)** CSp neuron labeled from the cervical spinal cord fired bursty episodes at 26°C, regularly at 36°C, and reversed back to bursty firing on decreasing the bath temperature back to 26°C. **(B)** Example of a typical DAP in a CSp neuron recorded at 26°C. The AP was triggered by slow ramp current injection. **(C)** Example of a CTh neuron that fired a spike doublet at 26°C and a single AP at 36°C. The spike doublet was triggered from the DAP following the initial AP at 26°C and only a small DAP was apparent at 36°C. On prolonged current injection the same neuron fired bursty episodes only at 26°C, and in a regular pattern at 36°C.

**FIGURE 5:**
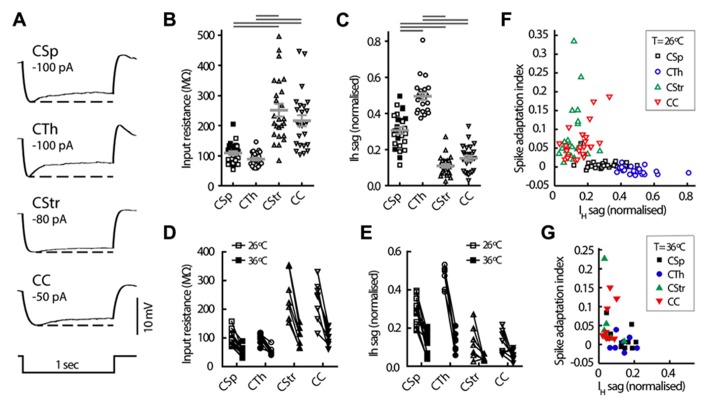
**Intrinsic electrophysiological properties of M1 layer 5 neurons grouped by projection target. (A)** Representative voltage responses to hyperpolarising current injection in the four cell groups at 26°**C**. Note the differences in size and kinetics of the *I*_H_ mediated depolarisation (or sag) seen after the peak hyperpolarisation, dashed line. Distribution of the cell input resistance **(B) **and sag potential **(C)** in the different cell groups at 26°C (means ±SEM indicated in gray, filled and open squares respectively for lumbar and cervical CSp neurons). Horizontal gray bars indicate group-wise differences at the 5% significance level (Kruskal-Wallis test and Dunn’s *post hoc* analysis). **(D, E)** Paired comparison of the same parameters at 26 and 36°C. Relationship between firing frequency adaptation and sag at 26°C **(F)** and 36°C **(G)**, showing the largest *I*_H_ sag in the CTh and CSp subgroups consistent with their regular firing behavior (see **Figure 3**) and the smallest *I*_H_ sag and robust adaptation in the CStr and CC neurons.

**FIGURE 6:**
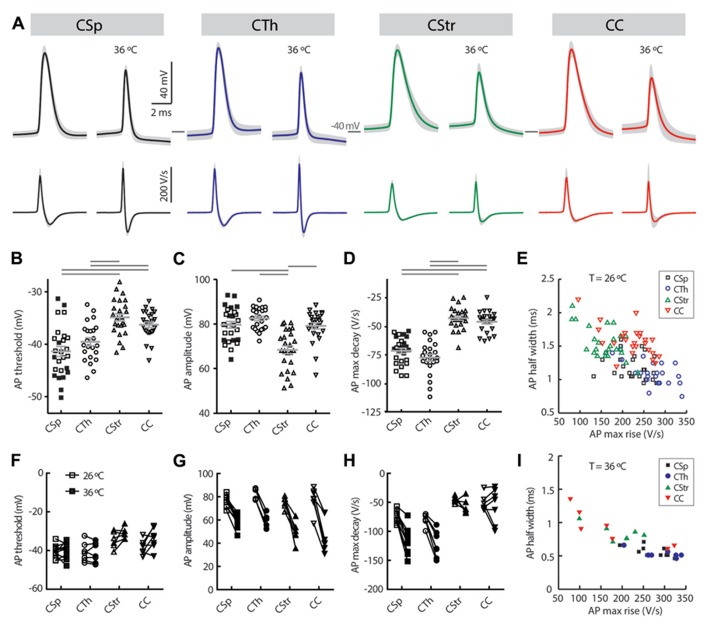
**Action potential waveform characteristics of M1 layer 5 neurons grouped by projection target. (A)** Average action potential waveforms (upper panel) and first derivatives of the waveform (lower panel) in the same neurons at 26°C (left) and 36°C (right; *n* = 10 CSp, 7 CTh, 7 CStr, and 7 CC neurons; a single AP was evoked in each neuron by a near threshold current ramp; SDs are indicated by gray shading). Distribution of the action potential threshold **(B)**, amplitude **(C)** and maximum decay **(D)** in the different groups at 26°C (means ±SEM indicated in gray, filled and open squares respectively for lumbar and cervical CSp neurons). Horizontal gray bars indicate group-wise differences at the 5% significance level (**B, D**, one-way ANOVA and Tukey’s *post hoc* test; **C**, Kruskal-Wallis test and Dunn’s *post hoc* analysis). **(F–H)** Comparison of the same parameters at 26 and 36°C for paired recordings. Relationship between action potential half width and maximum rise at 26°C **(E)** and 36°C **(I)**, highlighting the fastest rising shortest duration of the action potential in the CTh neurons.

**FIGURE 7:**
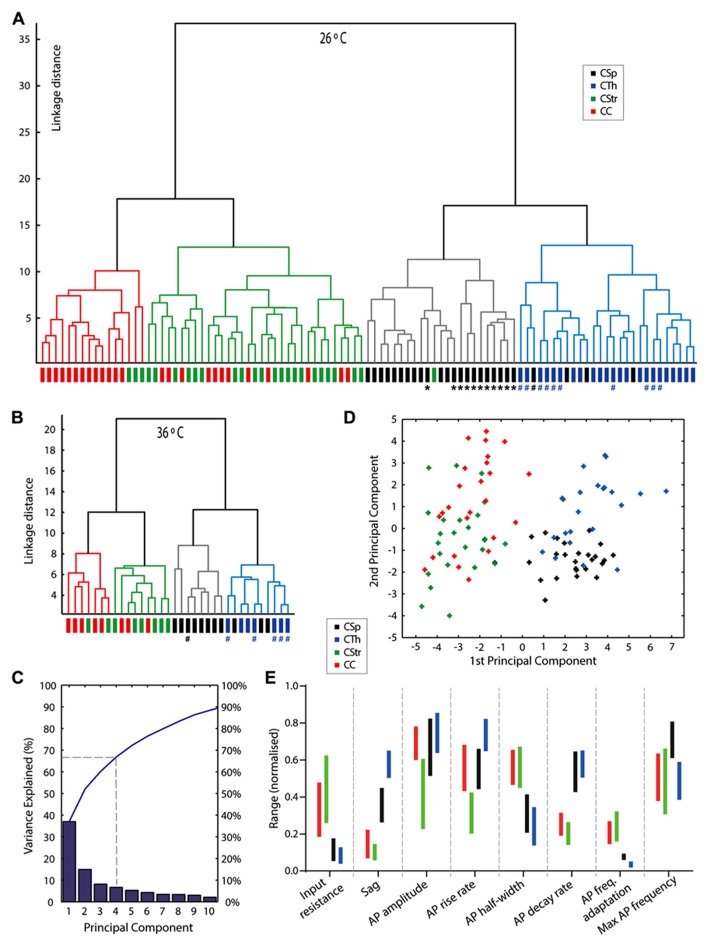
**An unsupervised cluster analysis reveals neuron grouping based on projection target.** Dendrogram plots of linkage distances from an unsupervised cluster analysis of 24 parameters for all cells at 26°C **(A)** and 36°C **(B)**. Four clusters were obtained in both data sets by setting the linkage threshold to 45% of the maximum linkage distance. Neurons that exhibited burst firing episodes at 26°C are marked with a hash #, 1 × CSp neuron and 10 × CTh neurons; at 36°C the same CSp neuron and 5 of the CTh neurons are marked since these neurons were recorded at both temperatures; importantly at 36°C none of these corticofugal neurones exhibited burst firing behavior, see **Figure 4A** for an example. CSp neurons projecting to the lumbar spinal cord are marked with an asterisk *. **(C)** Graph of the variances explained by the first ten components from a princial component analysis of the 26°C data. The blue line shows the cumulative variance. **(D)** Plot of scores for each cell from the first two principal components (CSp, black; CTh, blue; CStr, green; CC, red). See also**Table 4**. **(E)** Non-overlapping interquartile ranges (vertical bars colored to represent cell type) of electrophysiological parameters for each cell type helps provide an indication of the key parameter differences for the different projection neuron types. The data is normalized to the absolute range (min to max, zero to one) of all values encountered for each parameter from the different groups of cells.

We used electrophysiological recordings at 26°C from all the layer 5 neurons to identify key differences in their intrinsic properties and AP firing behavior. Since temperature influences electrophysiological parameters we also recorded a subset of neurons at both low and higher physiological temperature; we did this in order to provide a direct illustration of how a variety of parameters in defined cell types change with temperature, to validate any comparisons to *in vivo* conditions, and to ensure that the segregation of neuron types under different temperature conditions remained robust. Importantly, this latter comparison showed that projection target-dependent differences persisted at 36°C (**Tables 2,3**; **Figures 3–7**). As previously described by others in the somatosensory and prefrontal cortices (Hattox and Nelson, 2007; Otsuka and Kawaguchi, 2008; Hirai et al., 2012) the different projection neurons displayed varying degrees of spike frequency adaptation (**Figures 3A–C**). The CSp neurons fired APs at the fastest rate and with the most regular pattern. The appearance of an initial fast spike doublet, or burst, also seen in the CTh neurons, at 26°C disappeared at 36°C (**Figures 3A,E**). CTh neurons displayed AP firing frequency acceleration at both temperatures, whereas AP firing rate characteristically decreased over time in commissural CStr and CC neurons (**Figures 3A,B**). The adaptation index derived from the regression slope of successive ISIs proved to be an effective discriminator between neuron types (**Figure 3C**; **Table 2**), at both temperatures (two-way ANOVA with Tukey’s *post hoc* test, *p* < 0.001 for cell type, *p* = 0.33 for temperature; *n* = 11 CSp, 7 CTh, 7 CStr, and 8 CC neurons). 

**Table 2:** Electrophysiological properties of M1 layer 5 projection neurons (26°C).

	CSp (*n* = 26)	CTh (*n* = 23)	CStr (*n* = 25)	CC (*n* = 25)	Statistics^‡^
Membrane potential (mV)	-64.9 (-61.4, -67.3)	-68.0 (-63.9, -69.9)	-67.9 (-63.9, -71.3)	-69.9 (-67.8, -70.6)	b ^1^, c ^2^
Input resistance (MΩ)	111 (78, 132)	85 (71, 111)	221 (168, 330)	209 (136, 266)	b ^3^, c ^3^, d ^2^, e ^3^
Membrane Tau (ms)	27.7 (21.2, 30.9)	20.5 (16.1, 24.3)	33.0 (29.0, 40.4)	34.4 (30.3, 46.7)	a ^1^, c ^2^, d ^3^, e ^3^
Sag ^∫^	0.30 (0.23, 0.38)	0.49 (0.42, 0.53)	0.10 (0.07, 0.14)	0.17 (0.08, 0.20)	a ^1^, b ^3^, c ^2^, d ^3^, e ^3^
Post-train AHP (mV)	-7.9 (-6.8, -9.5)	-8.5 (-7.4, -9.4)	-3.8 (-2.6, -5.7)	-3.2 (-2.6, -4.6)	b ^3^, c ^3^, d ^3^, e ^3^
Log adaptation ^§^	3.56 ± 0.56	4.33 ± 0.14	2.45 ± 0.11	2.53 ± 0.09	a ^3^, b ^3^, c ^3^, d ^3^, e ^3^
Max mean inst freq (Hz)	39.2 (36.6, 45.8)	37.5 (31.3, 69.1)	39.4 (34.1, 44.7)	30.8 (26.5, 28.0)	c ^2^, e ^1^, f ^2^
Max frequency (Hz)	34.1 (31.2, 38.6)	26.0 (22.6, 30.4)	29.2 (19.6, 33.1)	25.2 (22.3, 32.0)	a ^2^, b ^1^, c ^2^
*I*_50% max frequency_ (pA)	273 (229, 333)	486 (323, 568)	214 (182, 289)	332 (211, 410)	a ^3^, d ^3^, e ^3^, f ^1^
Frequency gain (Hz/pA)	0.082 ± 0.002	0.069 ± 0.002	0.088 ± 0.007	0.082 ± 0.005	p = 0.056

‡ As for ***Table*1**.

∫ Sag potential of hyperpolarisation and cyclic nucleotide-activated current, normalized to peak potential.

§ Normalized data (absolute values of the natural Log of the adaptation index after adding a constant of 0.025 to each index to remove negative data values).

**Table 3:** Action potential waveform properties of M1 layer 5 projection neurons (26°C).

	CSp (*n* = 26)	CTh (*n* = 23)	CStr (*n* = 25)	CC (*n* = 25)	Statistics^‡^
AP threshold (mV)	-41.4 ± 1.0	-39.5 ±0.8	-34.9 ±0.7	-36.3 ±0.5	b^3^, c^3^, d^3^, e^1^
AP amplitude (mV)	79.6 (72.7, 85.6)	82.9 (77.9, 86.9)	68.1 (60.7, 76.5)	81.3 (76.3, 83.8)	b^3^, d^3^, f^3^
AP half-width (ms)	1.20 ±0.04	1.10 ±0.04	1.55 ±0.05	1.58 ±0.05	b^3^, c^3^, d^3^, e^3^
AP max rise (V/s)	222 ±8	272 ±9	159 ±8	221 ±10	a^3^, b^3^, d^3^, e^3^, f^3^
AP max decay (V/s)	-71.1 ±2.3	-77.1 ±3.1	-42.5 ±1.9	-45.7 ±1.8	b^3^, c^3^, d^3^, e^3^
mAHP amplitude (mV)	-12.3 (-11.0, -14.8)	-16.3 (13.4, -16.7)	-16.0 (-13.9, -17.9)	-15.8 (-13.4, -18.8)	a^3^, b^2^, c^2^

‡ As for ***Table*1**.

**Table 4:** Squared coefficients expressed as percentages for the first 10 principal components.

	PC1	PC2	PC3	PC4	PC5	PC6	PC7	PC8	PC9	PC10
RMP	0.41	8.63	0.03	0.99	0.28	19.54	5.33	34.86	3.93	0.02
Input resistance (Ri)	8.56	1.73	0.70	0.62	0.83	1.17	2.91	0.12	2.49	0.00
Tau	5.24	0.51	0.30	8.29	2.77	0.50	8.07	2.16	4.70	1.97
Post-train AHP	6.06	2.53	0.66	0.07	9.44	0.00	2.60	3.43	5.56	6.34
Sag	7.09	0.14	0.97	0.11	10.14	0.22	5.24	0.02	2.83	11.45
Bregma level	0.47	6.34	1.27	0.37	0.76	25.07	3.75	32.79	11.05	0.00
AP peak amplitude	3.00	2.77	13.41	12.63	0.00	3.97	5.52	1.53	0.00	1.32
AP half-width	7.52	0.03	2.74	5.11	3.69	0.00	0.06	0.07	0.82	4.92
AP max rise rate	4.87	4.14	12.50	1.12	0.38	1.31	9.70	1.82	0.24	0.02
AP max decay rate	8.78	0.07	5.20	0.28	2.49	0.69	0.25	0.04	0.72	1.40
AP threshold	2.85	1.98	4.40	16.19	2.33	0.21	10.33	0.00	2.24	11.53
mAHP	0.04	5.62	2.02	23.79	4.40	0.62	1.07	1.18	22.36	6.39
AP frequency adaptation	6.66	1.39	3.61	1.21	0.38	5.92	0.61	0.09	0.46	12.07
Soma size	4.84	0.97	6.16	4.35	1.67	5.43	0.05	0.04	10.85	19.75
Shaft width	7.38	2.35	1.87	2.65	0.23	0.40	0.97	0.03	0.47	4.15
Tuft hight	5.29	2.44	9.74	0.28	2.12	0.07	8.87	3.01	0.06	1.00
Tuft width	6.25	0.61	0.17	1.61	10.61	0.65	8.90	0.80	1.47	0.66
Apical dendrite height	4.51	2.20	7.85	4.58	11.03	0.16	0.03	0.33	3.92	1.40
Soma depth	2.39	2.23	5.47	3.69	30.78	0.13	0.52	1.32	1.33	4.24
Current at 50% max frequency	3.68	13.17	0.08	0.45	0.17	0.03	0.06	2.32	0.38	3.78
Max frequency	0.55	15.36	5.31	0.03	2.63	1.97	1.50	3.61	0.57	1.76
Max frequency gain	0.60	12.42	1.36	0.58	0.49	25.70	4.49	5.49	0.00	1.70
Max instantaneous frequency	0.65	10.31	4.73	6.13	0.08	0.82	14.83	0.29	0.43	3.51
Tuft origin	2.33	2.06	9.45	4.88	2.32	5.42	4.36	4.66	23.13	0.60
Sum	100.00	100.00	100.00	100.00	100.00	100.00	100.00	100.00	100.00	100.00
Sum of highlighted variables	58.29	51.27	52.95	60.90	72.00	70.31	60.69	67.64	67.38	54.80

*Important contributions are highlighted in sky blue (negative sign) and yellow (positive sign).*

Traditionally, cortical pyramidal neurons are classified as regular or burst firing types (Amitai and Connors, 1995). Most of the burst firing neurons encountered in this study were CTh neurons (*n* = 12/23), although we also encountered bursting in 4/15 CSp neurons projecting to the cervical spinal cord. None of the 11 lumbar CSp neurons exhibited bursts. Furthermore, we noted the absence of bursting at 36°C in all cells even though we encountered bursting in the same cells at 26°C (**Figures 3D,7A,B**), as exemplified by the extreme non-linearity of the instantaneous frequency plots. Temperature effects on bursting were reversible (2 of 2 neurons tested and an example is shown in **Figure 4**). These types of temperature effects on AP firing pattern have been described previously for M1 layer 5 pyramidal neurons from mature mice (Hedrick and Waters, 2011, 2012), suggesting that cell intrinsic bursting of corticofugal neurons recorded *in vitro* is induced at lower temperatures. In contrast, AP firing behavior in CStr and CC neurons was not influenced by temperature (**Figures 3A–E**).

The average AP firing rate in response to successively increasing depolarising current injections increased in a sigmoidal fashion. Fitting a sigmoidal function to the input – output relationship of individual neurons (**Figure 3F**) allowed us to estimate the maximum mean firing rate and the gain in frequency during the linear response phase. CSp neurons reached higher average firing rates compared to the other three groups at both 26 and 36°C (**Figures 3F,G**; **Table 2**, two-way ANOVA with Tukey’s *post hoc* test, *p* < 0.05 for cell type, *p* < 0.001 for temperature). CC neurons exhibited steeper frequency gains compared to CTh and CStr neurons when considering both temperatures (**Figure 3H**, two-way ANOVA with Tukey’s *post hoc* test, *p* < 0.01 for cell type, *p* < 0.001 for temperature). Collectively the differences in firing behavior suggest that a unique complement of voltage dependent conductances is expressed in each of the four projection neuron classes.

### SUBTHRESHOLD MEMBRANE PROPERTIES

To test for projection target specific signatures of voltage dependent conductances in the subthreshold voltage range to AP firing we assessed basic electrophysiological properties that generally differentiate between commissural and corticofugal neurons (Kasper et al., 1994; Christophe et al., 2005; Le Be et al., 2007; Dembrow et al., 2010; Sheets et al., 2011; Suter et al., 2012). Though CSp neurons were somewhat more depolarised at rest at 26°C (**Table 2**) the resting membrane potential did not differ between the cell types when measured also at 36°C (two-way ANOVA, *p* = 0.19). The input resistance of CStr and CC neurons was greater than that of CSp and CTh at both 26°C (Fig. 5B) and 36°C (two-way ANOVA, *p* < 0.001 for cell-type and temperature). Membrane time constants were similar across all groups at physiological temperature (grand mean = 20.5 ±0.2 ms, see also **Table 2**). The magnitude of the sag was largest in CTh neurons and more pronounced in CSp neurons than in CStr and CC neurons (**Figures 5A,C**; ****Table 2****). As with input resistance, the sag potential decreased overall at 36°C compared to 26°C (**Figure 6E**) but the cell group differences remained (two-way ANOVA, *p* < 0.001 for cell-type and temperature). Hence the magnitude of the sag further differentiated between CSp and CTh corticofugal neurons.

*I*_H_ is a pacemaker current that supports both regular and burst firing modes in neurons (Robinson and Siegelbaum, 2003). *I*_H_ mediated sag potential and spike frequency adaptation correlated inversely at both temperatures, so that CSp and CTh neurons with a large sag had very little spike adaptation while CStr and CC neurons with a smaller sag had high adaptation indices (**Figures 5F,G**). However, this correlation does not appear to have a causative relationship as *I*_H_ attenuation by noradrenergic modulation reported by others (Sheets et al., 2011) does not affect spike frequency adaptation in CSp neurons. Although hyperpolarising calcium-activated potassium currents have been associated with spike frequency adaptation in other neurons (Faber and Sah, 2005), the post-train AHP was noticeably larger in CTh and CSp neurons compared with CC and CStr neurons (**Table 2**), a distinguishing feature that persisted at both temperatures (two-way ANOVA with Tukey’s *post hoc* test, *p* < 0.001 for cell-type and temperature). Spike frequency adaptation or acceleration likely depends on the functional interaction of multiple conductances such as slow AHP and slowly inactivating Kv channels (Miller et al., 2008).

### ACTION POTENTIAL CHARACTERISTICS

Previous studies have used the AP waveform as an identifier for different projection neuron types in the rat visual (Mason and Larkman, 1990) and perirhinal cortex (Moyer et al., 2002), and in the mouse somatosensory (Hattox and Nelson, 2007) and motor cortex (Suter et al., 2012). Similarly, AP waveform characteristics varied markedly between layer 5 neurons (**Table 3**; **Figure 6**) at both recording temperatures. CTh and CSp neurons exhibited lower thresholds to generate an AP compared to CC and CStr neurons (**Figures 6B,F**; **Table 3**; two-way ANOVA of paired recordings, *p* < 0.001 for cell-type, *p* = 0.11 for temperature). CTh and CSp neurons exhibited faster APs than those of CC and CStr neurons (half width and maximum decay rates) at both temperatures (**Figures 6A,D,E,H,I**; **Table 3**; two-way ANOVA of paired recordings, *p* < 0.001 for cell-type and temperature). Increasing the recording temperature decreased the AP half width in all cell types (*p* < 0.001 each) while maximum decay rates were increased selectively in corticofugal neruons only (**Figure 6H**, two-way repeated measures ANOVA, *p* < 0.001 for CSp and CTh neurons, *p* > 0.05 for CStr and CC neurons, Bonferroni’s *post hoc* analysis).

In addition to the AP decay rate, its maximum rise rate also correlated well with its half-width (**Figures 6E,I**; *R*^2^ = 0.39 and 0.77 at 26 and 36°C, respectively). CTh neurons exhibited the fastest maximum rise rate of the AP and CStr neurons the slowest (**Table 3**) at both temperatures (two-way ANOVA with Tukey’s *post hoc* test, *p* < 0.001 for cell type, *p* = 0.15 for temperature). The AP amplitude also differed between cell groups. CStr neurons had the smallest amplitude APs (**Figure 6C**; **Table 3**). At 36°C CStr and CC neurons exhibited lower AP amplitudes compared to CSp and CTh neurons (two-way ANOVA of paired recordings, *p* < 0.01 for cell type, *p* < 0.001 for temperature;* p* > 0.05 for CSp versus CTh and CStr versus CC cells, Tukey’s *post hoc* test). CSp neurons uniquely exhibited the smallest mAHP following a single AP (**Table 3**, two-way ANOVA with Tukey’s *post hoc* test, *p* < 0.001 for cell type, *p* > 0.05 for temperature).

Corticofugal neurons and CTh neurons in particular, exhibited a pronounced depolarising afterpotential (DAP) following a spike (see **Figure 4B**) as highlighted by Hattox and Nelson (2007) for CTh and corticotrigeminal projection neurons in somatosensory cortex. We did not include the DAP as a quantitative variable because it depends on the relative strengths of the fast and mAHP and it could not be measured in neurons that fired an initial fast spike doublet in response to the near threshold current ramp injection (15 out of 23 CTh compared to 2 of 26 CSp neurons recorded at 26°C). An example of a spike doublet triggered by the DAP from a CTh neuron that also fired a burst at 26°C, but not at 36°C is shown in **Figure 4C**.

### CLUSTER ANALYSIS REVEALS FOUR SUBTYPES

Our results identified key differences between cell groups according to axonal projection target (Hattox and Nelson, 2007) but it is possible that we introduced some bias by first grouping cells according to their projection target. We therefore performed an unsupervised cluster analysis of 24 electrophysiological and morphological parameters (see Materials and Methods) to test how neurons segregated without prior specification of projection target. As seen in **Figures 7A,B** the cells separated into 4 groups and two main groups with a good match to projection target. An important caveat here is that uncertainties, or biases, can arise in this type of cluster segregation if different projection neuron types share collaterals. As we can see, CSp and CTh versus CStr and CC neurons clearly segregated into two main groups, but within these there was some overlap beween CSp and CTh, and CStr and CC neurons, respectively. This probably arose from the fact that some commissural CStr neurons also send a collateral to the cerebral cortex (Wilson, 1987). An explanation for the overlap between CSp and CTh neurons lies with the likelihood that some CSp neurons also send a collateral to the thalamus. To fully determine the extent of such ambiguity caused by overlapping projections would require targeting from multiple sites with distinct colors. The cluster analysis also revealed two sub groups within the CSp neurons and this seemed to relate to whether they were labeled from either the lumbar or the cervical spinal cord (see **Figure 7A**, asterisks label lumbar neurons). Selective labeling of lumbar CSp neurons (compared with cervical CSps) is possible since, at least in the cat, lumbar neurons do not possess collaterals in the cervical spinal cord (Shinoda et al., 1986). Only cervical CSp neurons exhibited bursting behavior, as previously described by Tseng and Prince (1993), and also shown in **Figures 7A,B** by a hash label, lumbar CSp neurons did not exhibit bursting behavior.

It was important to confirm that the cluster-based segregation persisted at physiological temperatures. Although linkage distances were compressed at 36°C compared with 26°C the salient features of both clusters persisted at 36°C, hash labels in **Figure 7B** indicate cells that exhibited bursts at 26°C but not at 36°C.

### PRINCIPAL COMPONENTS ANALYSIS

A PC analysis using the same variables as for the cluster analysis revealed that the first two PCs explained just over 50% of the variance, four PCs explained approximately two-thirds, and ten components explained 90% of the variance (**Figure 7C**). We identified variables that contributed most to each PC from the squared coefficients of each variable (**Table 4**). The first PC contained several parameters that made similar contributions, with input resistance, sag, AP half width, AP maximum decay, firing frequency adaptation, post-train AHP amplitude, shaft width, and tuft width contributing 58% to the first PC coordinate space. The AP firing rate parameters dominated the second PC coordinate space. A plot of the first two PC scores (**Figure 7D**) shows that the first PC already segregated the neurons into two main groups but considerable overlap remained beween CSp and CTh, and CStr and CC neurons in the second dimension. Most of the remaining variables significantly contributed to the next two PCs. AP amplitude and maximum rise rate, apical dendrite height, and tuft height and origin contributed 53% to the third PC, while membrane time constant, AP threshold, amplitude and size of the post-train AHP contributed 61% to the fourth PC (**Table 4**).

In **Figure 7E**, we aim to illustrate how the different electrophysiological parameters identified in the PCs analysis can be used to discriminate individual neuron types. In order to provide the experimenter with an idea of how the different parameters vary amongst the cells we first show the interquartile range normalized to the range of the data (maximum to minimum, 1 to 0, for all values of each electrophysiological parameter across all cell types). Little or no overlap of these normalized interquartile ranges provides an indication of the parameters that are best suited to discriminate the different cell types if the experimenter is blind to the projection target. As seen in **Figure 7E**, the sag, AP waveform parameters and firing properties, including adaptation proved to be the most useful discriminators.

## DISCUSSION

### DISTINCT SUBGROUPS OF M1 LAYER 5 PROJECTION NEURONS

Our electophysiological and morphological phenotyping identified at least four different types of pyramidal neurons within the principal output layer 5 of M1. A limited set of up to 8 parameters including dendritic shaft width, Ri, sag, AP half width, and spike frequency adaptation allowed reliable identification of the two main groups of projection neurons; corticofugal (main axon projecting toward the spinal cord) sometimes called PT-type, and commisural (main axon projecting across the corpus callosum to the cerebral cortex and/or the striatum) sometimes called IT-type neurons as also seen in other cortical regions (Amitai and Connors, 1995; Reiner et al., 2003; Christophe et al., 2005; Hattox and Nelson, 2007; Groh et al., 2010; Suter et al., 2012). We show that in M1 the application of an extended set of electrophysiological and morphological parameters further divided neurons into two sub-groups that matched their projection targets and allowed clear segregation of CSp and CTh neurons in the corticofugal group and CC and CStr neurons in the commissural group.

### MORPHOLOGY

Morphological parameters revealed clear differences between large soma, thick shafted, large tufted corticofugal neurons and small soma, slender shafted, small tufted commissural neurons (**Figure 2**), as previously described (Chagnac-Amitai et al., 1990; Mason and Larkman, 1990). The larger size of the CSp and CTh apical dendritic tuft in layer 1 implies that these neurons are more heavily influenced by inputs into this layer. The Sholl analysis in **Figure 2** also identified clear differences between the CTh and CSp neurons where the latter exhibited the largest and most complex dendritic tree. Dendrites from both groups were spiny (data not shown) and CTh neurons exhibited a wider apical tuft width relative to tuft height than CSp neurons.

CTh neurons in M1 shared many of the morphological features of CTh neurons in mouse somatosensory cortex labeled from the posteromedial thalamic nucleus, except that here the CTh neurons in M1 located superficially in layer 5a compared with lower layer 5 (layer 5b) in somatosensory cortex (Hattox and Nelson, 2007). The superficial location of M1 CTh neurons proved to be a reliable discriminator from the CSp neurons in L5b (**Figures 1F,G,2**). This is of physiological relevance since M1 receives sub-layer specific input. CTh neurons in upper layer 5 of M1 are likely to receive direct synaptic input from sensory brain regions whereas CSp and other layer 5b neurons are preferentially activated from the frontal cortex (Anderson et al., 2010; Mao et al., 2011). A recent study in the frontal cortex colabeled CTh neurons with corticopontine neurons in layer 5a (Hirai et al., 2012) so it is possible that the CTh neurons identified here in M1 may be brainstem projecting neurons that also send a collateral to the thalamus (see also schematic in **Figure 1A**).

Corticocortical and corticostriatal neurons exhibited largely similar morphologies and we noted that the apical dendrites of a minority of commissural CC and CStr neurons did not reach layer 1 (8 of 39 neurons). Apical dendrites of commissural CC neurons in the visual cortex typically do not reach layer 1 (Kasper et al., 1994), although commissural neurons in somatosensory and frontal cortices mostly do (Hattox and Nelson, 2007; Otsuka and Kawaguchi, 2011). Our results imply additional diversity within this group in M1, as also suggested from a retrograde tracing study in mouse barrel cortex where some CC neurons exhibited non-tufted dendrites that did not reach the pia whereas others possessed slender tufted dendrites that reached into layer 1 (Larsen et al., 2007).

### ACTION POTENTIAL FIRING PATTERN AND WAVEFORM

Cortical pyramidal cells traditionally have been differentiated by their bursting or regular firing pattern and the degree of the spike frequency accommodation (Connors et al., 1982; McCormick et al., 1985; Chagnac-Amitai et al., 1990; Agmon and Connors, 1992). The steady phase of the spike frequency adaptation following a faster initial adaptation rate proved a useful differentiator between cortical cell types (Agmon and Connors, 1992; Cho et al., 2004a; Hattox and Nelson, 2007; Llano and Sherman, 2009; Suter et al., 2012). In our sample of M1 neurons, spike frequency adaptation readily differentiated between cells in that CStr and CC neurons exhibited fast and progressive slowing of their firing rate whereas the CSp and CTh neurons did not (**Figure 3**). The CSp neurons fired APs with the highest frequency and also with a higher gain than CTh neurons implying that of all the neurons in M1 they are best suited to converting synaptic input to reliable output. The fast, highly regular firing behavior of the CSp neurons is consistent with their role as “driver”neurons and with their need for a high safety factor for AP conduction (Kuznetsova et al., 2010). CTh neurons contrasted with CSp neurons by their ability to fire accelerating APs. A previous study (Miller et al., 2008) in mouse cortex neurons projecting to the brainstem attributed this phenomenon to their selective expression of slowly inactivating Kv1 potassium channels. Based upon this accelerating phenotype, the CTh neurons in the present study may be brainstem-projecting neurons that also send a collateral to the thalamus (see **Figure 1A** and discussion of their overlap with corticopontine neurons above). Interestingly, corticopontine layer 5 projection neurons in the medial prefrontal cortex also demonstrate accelerating APs (Dembrow et al., 2010).

The AP waveform proved to be another useful differentiator (**Figure 6**) between the groups. The large CSp and CTh neurons, particularly the CTh neurons exhibited the largest amplitude, fastest AP waveform consistent with their large soma size and their fast conducting axons, presumably to ensure accurate and reliable communication over long distances (Sakai and Woody, 1988; Vigneswaran et al., 2011; Suter et al., 2012). CC and CStr neurons exhibited slower, smaller amplitude APs, perhaps because their projection targets are closer, whilst the CStr group exhibited the broadest APs of all (**Figure 6E**). Perhaps this broadness facilitates calcium influx to lower the threshold for spike timing-dependent synaptic plasticity (Markram et al., 2011).

### CELL-INTRINSIC BURST FIRING

Do intrinsically bursting neurons form a distinct cell class in M1? Like Hedrick and Waters (2011, 2012) in neocortical neurons, we encountered bursting phenotypes in corticofugal neurons only at lower recording temperatures (**Figure 3D**), with bursts most often seen in CTh neurons (see **Figure 4**). All neurons with a bursting phenotype at the lower temperature converted to a regular firing pattern at higher current injections, as in rat M1 (Tseng and Prince, 1993). Nevertheless, bursting neurons have also been described in layers 2–6 of cat M1 (Chen et al., 1996) at physiological temperatures. In other regions of the cortex burst firing neurons are present in upper layer 5 of guinea pig cingulate and somatosensory cortices (McCormick et al., 1985) and lower layer 5 of mouse and rat somatosensory and visual cortices (Chagnac-Amitai et al., 1990; Mason and Larkman, 1990; Agmon and Connors, 1992), and throughout layers 2–6 *in vivo* where their switch between regular and burst firing modes could underlie different vigilance states (Steriade et al., 2001). However, since burst firing was absent at physiological temperatures in M1 using it to group corticofugal neurons into distinct classes was not justified. Indeed, the cluster analysis at 26°C did not put CTh burst firing neurons into a distinct dendrogram branch consistent with the idea that burst firing is not a unique characteristic of M1 corticofugal neurons but an alternative firing mode of otherwise regular firing neurons. The switch between regular and burst firing modes may be regulated by the activation state of persistent sodium channels in the first node of Ranvier (Kole, 2011) as well as voltage-sensitive conductances that influence coupling between axo-somatic and dendritic compartments (Larkum et al., 1999; Williams and Stuart, 1999; Bekkers and Häusser, 2007). We might speculate that a differential temperature sensitivity of conductances influencing the somato-dendritic coupling in M1 corticofugal neurons could explain the switch between firing modes at 26 and 36°C. Convergent synaptic input to distal apical tuft dendrites may also influence burst firing in layer 5 neurons (Xu et al., 2012), although this is unlikely to have been a factor here as we blocked synaptic transmission throughout.

### ARE THERE SUBTYPES OF CSp NEURONS?

We noted that CSp neurons labeled from the lumbar spinal cord were found in M1 more posterior to Bregma (hindlimb) whilst a good proportion of the cervical cord projecting neurons located anterior to Bregma (forelimb). In addition, electrophysiological and morphological parameters meant that these lumbar and cervical CSp neurons segregated into different subgroups within the CSp cluster and exhibited subtly different electrophysiological parameters. This result suggests distinct intrinsic phenotypic differences among forelimb and hindlimb CSp projection neurons, perhaps because they reside within different regions and microcircuits of M1 and/or are influenced by distinct modulatory inputs. There is the possibility that cervical injections also label lumbar CSp neurons. However, this seems unlikely since, at least in the cat, the lumbar CSp neurons do not have collaterals in the cervical spinal cord (Shinoda et al., 1986). Our results therefore support the presence of additional diversity amongst M1 CSp neurons based upon their unique projection targets.

### *I*_H_ Sag

All M1 layer 5 projection neurons exhibited a “sag”potential indicative of the activation of the hyperpolarisation-activated inward current *I*_H_ (Christophe et al., 2005; Sheets et al., 2011), but it was most prominent in the CTh neurons both in amplitude and time course. Given the ability of *I*_H_ to dampen excitatory postsynaptic potentials in CSp neurons (Sheets et al., 2011) we would expect that neuromodulatory influences on *I*_H_ gating (Robinson and Siegelbaum, 2003) will shape synaptic processing by CTh neurons to an even greater extent. In addition *I*_H_ gives rise to distinct membrane resonance properties in commissural and corticofugal neurons (Dembrow et al., 2010).

### PHENOTYPIC FINGERPRINTING WITHIN LAYER 5 OF M1

Our approach has addressed and refined the previously acknowledged basis for projection neuron heterogeneity within layer 5 of M1. Resolving this diversity in M1 has relied upon a combination of electrophysiological and morphological parameters, the position of each neuron within layer 5 and their projection target, and was upheld by an unbiased segregation with the aid of a cluster analysis. Using the parameters highlighted by the PCs analysis (**Figure 7D**; **Table 4**) we also provide a practical guide to the electrophysiological parameters best suited to discriminate individual neuron types if the experimenter is blind to the projection target (summarized in **Figure 7E**). Nevertheless, projection target is still an important determinant of cell type identity in M1 but we acknowledge that ambiguities can arise if neurons have overlapping projection targets, and under these circumstances it is hard to resolve whether the electrophysiological properties are also distinct. To fully resolve projection neuron identity, double or triple labeling of neurons from multiple overlapping sites with associated electrophysiological identification is required (Otsuka and Kawaguchi, 2011; Sohur et al., 2012). Additional criteria (where known) such as local axonal ramification (Larsen et al., 2007), pre- and post-synaptic properties (Schubert et al., 2006; Kiritani et al., 2012), specific gene expression (Christophe et al., 2005; Molyneaux et al., 2007; Schmidt et al., 2012), or responsiveness to metabotropic neurotransmitters (Dembrow et al., 2010; Schmidt et al., 2012) can also help refine group clusters. In summary, a CSp neuron is the largest of the M1 neurons (soma, shaft and dendrites) that sits deepest in the layer, has a low input resistance and modest sag, and fires the most regular, highest frequency and very fast APs. Subtle differences also exist between lumbar and cervical projecting CSp neurons. CTh neurons also have large soma, shaft and dendrites but are found more superficially within layer 5 of M1. These neurons also possess the largest and fastest sag, and the fastest and narrowest APs that accelerate during prolonged depolarisation. In contrast, the smaller CC and CStr neurons have a higher resistance and exhibit wide APs that slow in frequency during a prolonged depolarisation. Those neurons with a slow rising AP of small amplitude are most likely CStr neurons. In contrast the smaller neurons located more superficially in layer 5a that require higher current levels to fire at half the maximal frequency are most likely CC neurons.

The generation of this set of four phenotypic fingerprints based upon key electrophysiological and morphological parameters provides a valid way to identify the major layer 5 projection neurons in mouse M1 (**Figure 7**). Hallmark differences between these projection neurons imply the need to communicate unique information to specialized downstream structures (Theyel et al., 2010; Turner and Desmurget, 2010; Morita et al., 2012; Palmer et al., 2012; Quallo et al., 2012). As we rely more and more on molecular markers to identify subtypes of neurons (Molyneaux et al., 2007), the phenotypic approach presented here will provide a useful and complementary tool to help further our continuing quest to untangle the motor cortex.

## Conflict of Interest Statement

The authors declare that the research was conducted in the absence of any commercial or financial relationships that could be construed as a potential conflict of interest.
